# Physiological Level Production of Antigen-Specific Human Immunoglobulin in Cloned Transchromosomic Cattle

**DOI:** 10.1371/journal.pone.0078119

**Published:** 2013-10-24

**Authors:** Akiko Sano, Hiroaki Matsushita, Hua Wu, Jin-An Jiao, Poothappillai Kasinathan, Eddie J. Sullivan, Zhongde Wang, Yoshimi Kuroiwa

**Affiliations:** 1 Kyowa Hakko Kirin, Co., Ltd., Chiyoda-ku, Tokyo, Japan; 2 Sanford Applied Biosciences L.L.C., Sioux Falls, South Dakota, United States of America; 3 Department of Animal, Dairy and Veterinary Sciences, Utah State University, Logan, Utah, United States of America; 4 Trans Ova Genetics, Sioux Center, Iowa, United States of America; 5 Hematech, Inc., Sioux Falls, South Dakota, United States of America; Michigan State University, United States of America

## Abstract

Therapeutic human polyclonal antibodies (hpAbs) derived from pooled plasma from human donors are Food and Drug Administration approved biologics used in the treatment of a variety of human diseases. Powered by the natural diversity of immune response, hpAbs are effective in treating diseases caused by complex or quickly-evolving antigens such as viruses. We previously showed that transchromosomic (Tc) cattle carrying a human artificial chromosome (HAC) comprising the entire unrearranged human immunoglobulin heavy-chain (h*IGH*) and kappa-chain (h*IGK*) germline loci (named as κHAC) are capable of producing functional hpAbs when both of the bovine immunoglobulin mu heavy-chains, b*IGHM* and b*IGHML1*, are homozygously inactivated (double knockouts or DKO). However, B lymphocyte development in these Tc cattle is compromised, and the overall production of hpAbs is low. Here, we report the construction of an improved HAC, designated as cKSL-HACΔ, by incorporating all of the human immunoglobulin germline loci into the HAC. Furthermore, for avoiding the possible human-bovine interspecies incompatibility between the human immunoglobulin mu chain protein (hIgM) and bovine transmembrane α and β immunoglobulins (bIgα and bIgβ) in the pre-B cell receptor (pre-BCR) complex, we partially replaced (bovinized) the hIgM constant domain with the counterpart of bovine IgM (bIgM) that is involved in the interaction between bIgM and bIgα/Igβ; human IgM bovinization would also improve the functionality of hIgM in supporting B cell activation and proliferation. We also report the successful production of DKO Tc cattle carrying the cKSL-HACΔ (cKSL-HACΔ/DKO), the dramatic improvement of B cell development in these cattle and the high level production of hpAbs (as measured for the human IgG isotype) in the plasma. We further demonstrate that, upon immunization by tumor immunogens, high titer tumor immunogen-specific human IgG (hIgG) can be produced from such Tc cattle.

## Introduction

Immunotherapy with human polyclonal antibodies (hpAbs) or immunoglobulins (hIgs) prepared from the plasma of normal and convalescing human donors have been shown to be an effective treatment for several human diseases [1]. For example, hpAbs derived from the plasma donated from the general population have been widely used to treat autoimmune and immunodeficiency diseases [2], and hpAbs from recovering patients or pre-vaccinated individuals are effective in treating infections caused by the same pathogens [3,4]. Human polyclonal antibodies are also used in medical emergency crisis situations such as severe acute respiratory syndrome (SARS) outbreaks [5]. However, the availability and applications of hpAbs are currently limited. A paucity of voluntary human donors has resulted in a severe shortage of hpAbs, and donor-patient disease transmission concerns as well as a lack of reproducibility restrict their usage [6].

To address the medical demand for hpAbs, different alternatives have been explored to substitute or even replace human plasma-derived hpAbs. For example, IgG or Fabs (fragments of antigen binding), prepared from the plasma of immunized horses or sheep has been widely used to treat severe envenomation resulting from snake and spider bites [7,8]. However, animal derived IgG tends to cause immune reactions from treated patients. For example, it has been reported that rabbit derived anti-thymocyte globulin (ATG) causes serum sickness in patients in several clinical applications [9,10]. Recently, an *in vitro* antibody production system based on recombinant DNA and mammalian cell culture technologies has been in the development by Symphogen A/S [11]. This approach tries to mimic polyclonal nature of humoral immune response by producing mixtures of monoclonal antibodies (mAbs) that recognize multiple epitopes of an antigen. Such an approach, if successful, has the potential to produce antibody mixtures in large quantities within a well defined system, allowing for improved reproducibility and elimination of the risks associated with human plasma-derived hpAbs. However, these antibody mixtures do not fully take advantage of the vastness of antibody diversity generated by natural immune responses. Additionally, as pre-defined antigens are needed to identify the mAbs and a lengthy process is needed to engineer cell lines expressing the recombinant mAbs, this system may not be useful for treatment of diseases in which antigens are not well characterized, such as in autoimmunity, nor in dealing with sudden outbreaks of infectious diseases such as the 2002 SARS epidemic [5]. 

To harness the power of natural humoral immune response not only for its unparalleled diversity but also for its capability to respond rapidly after antigen exposure, we have been developing a transchromosomic (Tc) bovine system that quickly produces diverse hpAbs in large quantities [12]. Previously, we reported the generation of Tc cattle carrying a human artificial chromosome (HAC) comprising the entire unrearranged germline loci of human immunoglobulin heavy-chain (h*IGH*) and kappa light-chain (h*IGK*) with homozygous double knockouts (DKO) of the two bovine Ig mu heavy-chain loci (bIGHM^-/-^ and bIGHML1^-/-^) [12]. In these cattle, the production of serum hpAbs, as measured for its hIgG isotype, was only up to 1.5 g/l which is significantly lower than the physiological serum IgG levels of approximately 5-10 g/l in normal humans. The level of fully hIgG (hIgG kappa; hIgG/hIgκ) was even lower, at only approximately 0.65 g/l [12]. Further analysis of the Tc cattle showed suboptimal development of B cells, indicating that the low hIgG production in these Tc cattle is the result of poor B cell development [12].

**Figure 1:**
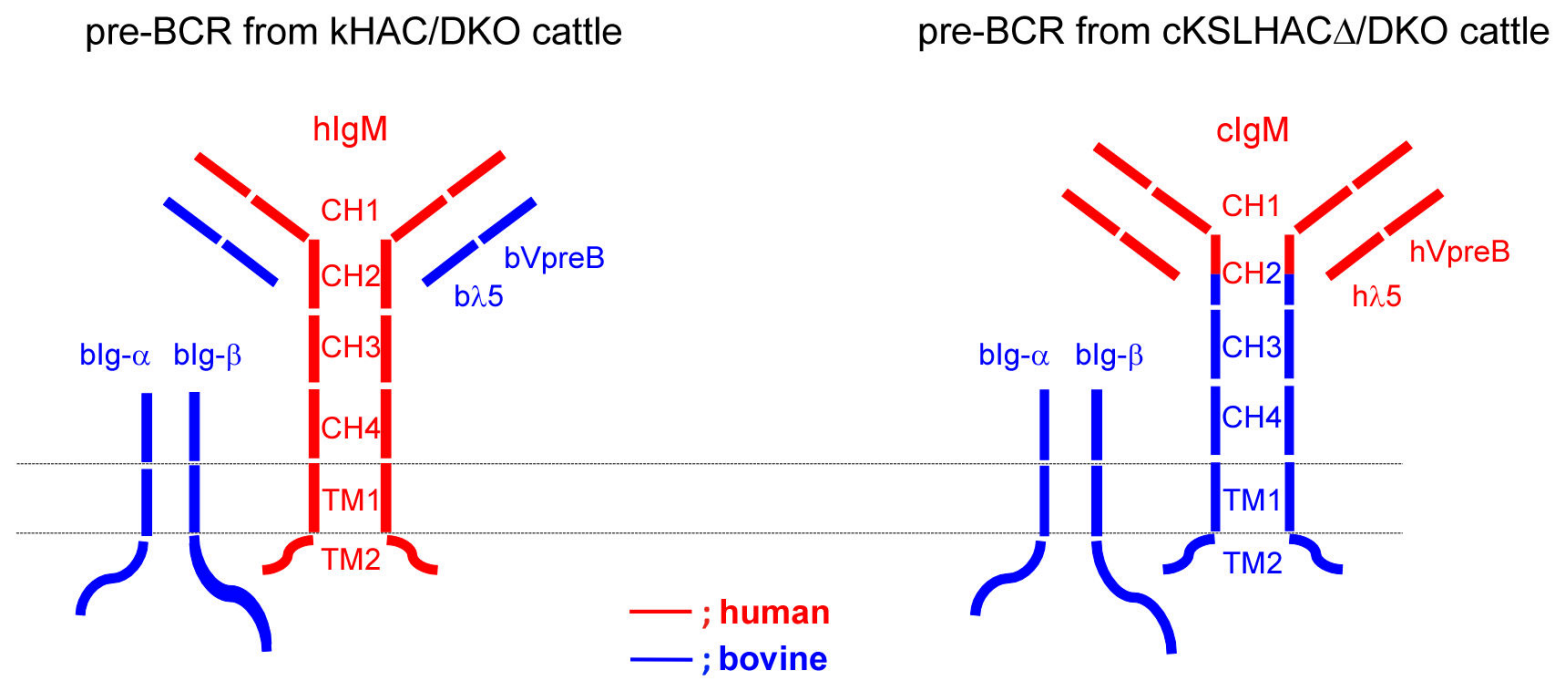
Diagrams of pre-BCRs in κHAC/DKO and cKSL-HACΔ/DKO cattle. Protein molecules/domains encoded by human genes are drawn in red while bovine in blue. The dotted double-lines represent B cell membrane.

The pre-B cell receptor (pre-BCR), comprising of IgM and invariant surrogate light-chains VpreB and lambda5 (λ5), plays critical roles for pre-B cell proliferation and maturation during B lymphogenesis [13]. It transmits B cell proliferation and maturation signals to the nucleus through its interaction with the B cell transmembrane proteins α and β immunoglobulins (Igα and Igβ) [14-16]. We reasoned that the poor B cell development and low hIgG production in the previously produced Tc cattle could be due to a compromised function of pre-BCR, as in the HAC/DKO cattle the bIgM in the pre-BCR complex is replaced by hIgM. Specifically, we speculated that, due to the difference in protein sequences between human and bovine IgMs, hIgM may not interact with bovine surrogate light-chains effectively; similarly, the functional interaction between the CH-TM domains of hIgM with the bovine Igα and Igβ proteins for transmitting the cellular signals from a pre-BCR to the nucleus may also be compromised. Therefore, in an effort to improve B cell development and hIgG production in Tc cattle, we sought to enhance pre-BCR function by engineering a new HAC into which, in addition to the h*IGH*, h*IGK* and h*IGL* chromosome loci that carry the entire human immunoglobulin gene repertoire, the human VpreB (h*VPREB1*) and λ5 (h*IGLL1*) genomic loci from human chromosome 22 (hChr22) was incorporated, and part of CH and TM domains, CH2-TM, of h*IGHM* gene, was replaced by the corresponding bovine gene sequence (bovinization of the CH2-TM domains of h*IGHM*). As IgM interacts with surrogate light-chains and Igα/Igβ via its variable (V) and CH-TM domain, respectively, such modifications would restore the natural intra-species protein-protein interactions in the pre-BCR (Figure 1). We also envisioned that the bovinized CH-TM domain would also improve the functionality of hIgM in interacting with bovine B cell proteins that are involved in B cell activation and proliferation. Our results showed that DKO Tc cattle carrying the modified HAC have greatly improved B cell development and produced up to 9 g/l of hIgG in the plasma. Through immunization studies, we further demonstrated that these Tc cattle are responsive to immunization with the tested tumor antigens and produce high titer tumor-specific hIgG. 

## Results

### Modification hChr14 for HAC Construction

#### 1. hChr14 modification

**Figure 2:**
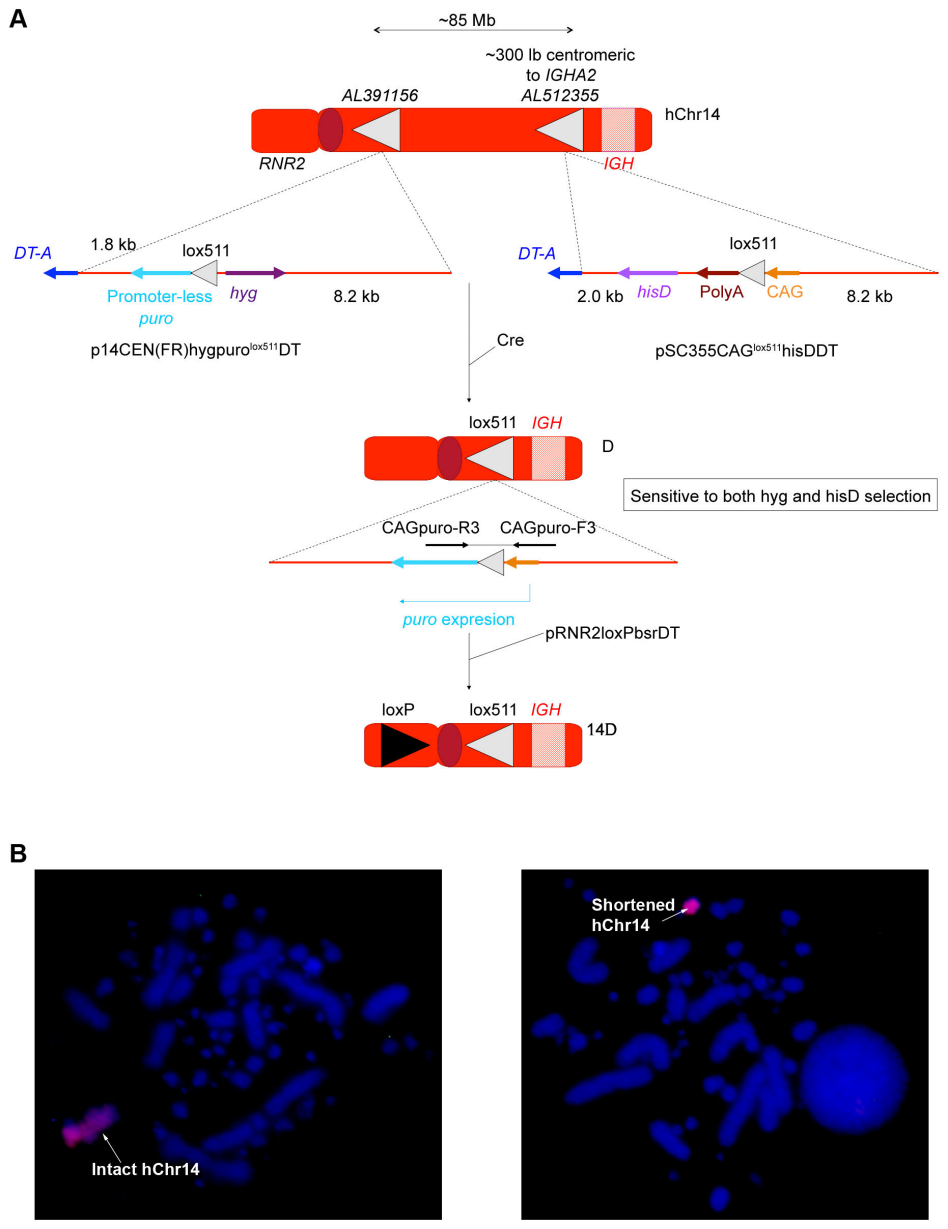
Modification of hChr14. (A) A lox511 sequence along with the promoter-less *puro* cassette was integrated at the AL512355 locus with gene targeting vector p14CEN(FR)hygpuro^lox511^DT, and a lox511 sequence along with a CAG promoter and a hygromycin (hyg) selection cassette was integrated at locus AL512355 with gene targeting vector pSC355CAG^lox511^hisDDT. Following Cre expression, the ~85 Mb genomic sequence was removed rendering *puro* expression. A loxP sequence and a GFP reporter cassette was then integrated at the *RNR2* locus to generate 14D. (B) FISH analysis of a DT40 clone, 14D, containing the correctly modified hChr14.

For deleting the non-immunoglobulin genes on hChr14, intact hChr14 was transferred to chicken DT40 cells by using Micro-cell Mediated Chromosome Transfer (MMCT) for chromosomal modification as described previously [17]. We employed a Cre/LoxP mediated site-specific recombination strategy to delete the DNA sequences on hChr14 that are irrelevant to immunoglobulin locus. Specifically, we integrated two lox511 sequences into hChr14, one at the *AL512355* locus (about 300 kb centromeric to the h*IGH* locus) and the other at *AL391156* locus (about the 85 Mb centromeric to the *AL512355* locus), through homologous recombination for deleting the 85 Mb sequences on hChr14 between these two loci (Figure 2A). In order to facilitate the identification of the correctly deleted DT40 cell clones, we also integrated a CAG promoter and a hisD selection cassette along with the lox511 sequence at locus *AL512355* and the promoter-less puromycin (puro) gene along with the second lox511 sequence and a hygromycin selection cassette at locus *AL391156*, respectively (Figure 2A; for the details, see Materials and Methods). Such strategy ensured that only the correctly deleted hChr14 acquired puromycin resistance and was sensitive to hygromycin and histidinol (*hisD*) (Figure 2A). Post electroporation of the DT40 cells with a Cre-expressing vector; the correctly deleted colonies were screened by genomic PCR and confirmed by fluorescence in situ hybridization (FISH) (data not shown). To prepare the correctly shortened hChr14 (labeled D) as one of the building blocks for the construction of the cKSL-HACΔ, it was further modified by integrating a loxP sequence and a GFP reporter cassette at the *RNR2* locus as described in Materials and Methods and previously [12]. Through extensive genomic PCR analysis (data not shown) and FISH (Figure 2B), a DT40 clone, 14D, was confirmed to have the loxP integration at the desired locus and selected for the bovinization of the CH2-TM2 domain of hIgM (see below).

#### 2. Bovinization of hIgM CH2-TM Domain

**Figure 3:**
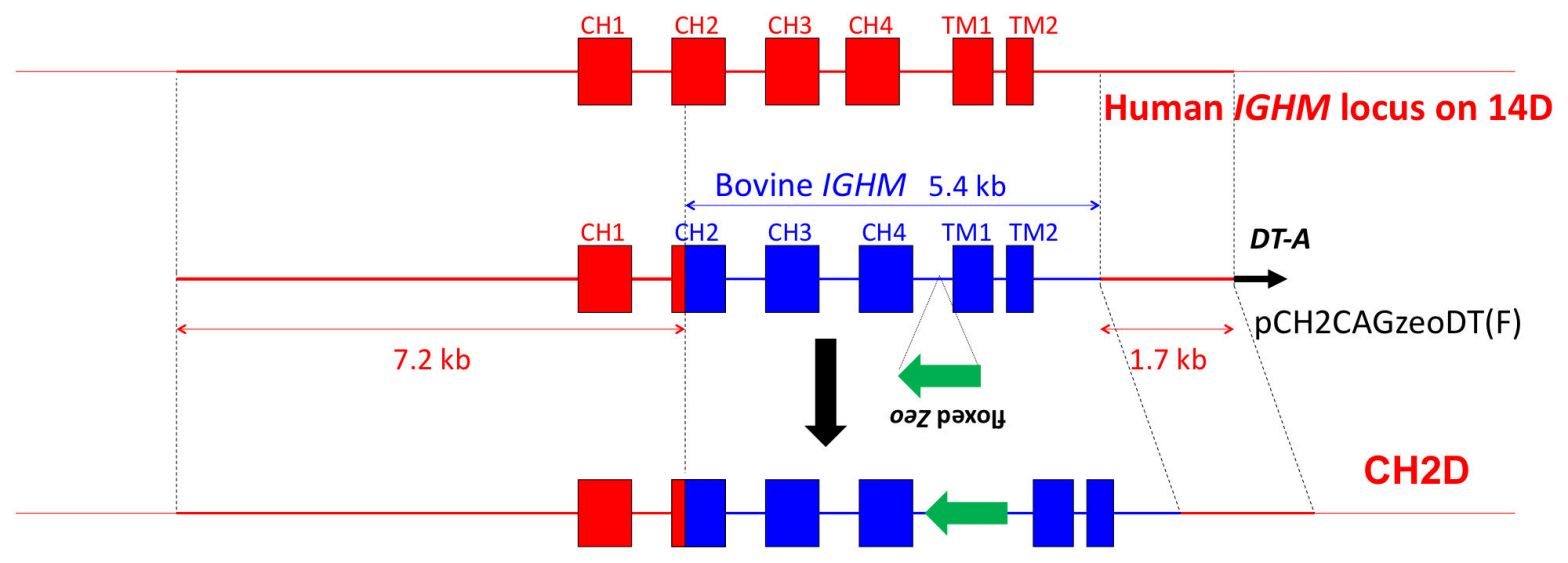
Bovinization of hIgM CH2-TM Domain. **The upper line shows the genomic configuration of the CH 1-TM2 domain of hIgM, the middle line shows the gene targeting vector pCH2CAGzeoDT, and the bottom line depicts the modified CH2-TM2 domain of hIgM in a DT40 cell clone, CH2D4**.

In order to improve the functional interactions between the hIgM and bIgα/Igβ proteins in the pre-BCR, as well as the overall functionality of hIgM in Tc bovine B cells, we constructed a gene-targeting vector to bovinize the CH2-TM2 domain of hIgM that is involved in interacting with bIgα/Igβ [18]. The bovine genomic DNA used for the gene targeting vector construction were cloned from an isogenic bovine genomic phage library (see Materials and Methods). We employed a positive and negative selection for this gene targeting event: a zeocin (*Zeo*) expression cassette flanked by loxP sequences (floxed-Zeo; for eventually removing of the Zeo cassette from the bovine genome) was inserted into the intron between CH 4 and TM1 for positive selection and a dT-A selection cassette was added outside of the 3’-homologous arm for negative selection (Figure 3). A DT40 cell clone, CH2D4, was confirmed by extensive genomic PCR genotyping experiments (data not shown) to carry the correctly targeted 14D and was selected for the final HAC construction.

### Modification of hChr22 for HAC Construction

**Figure 4:**
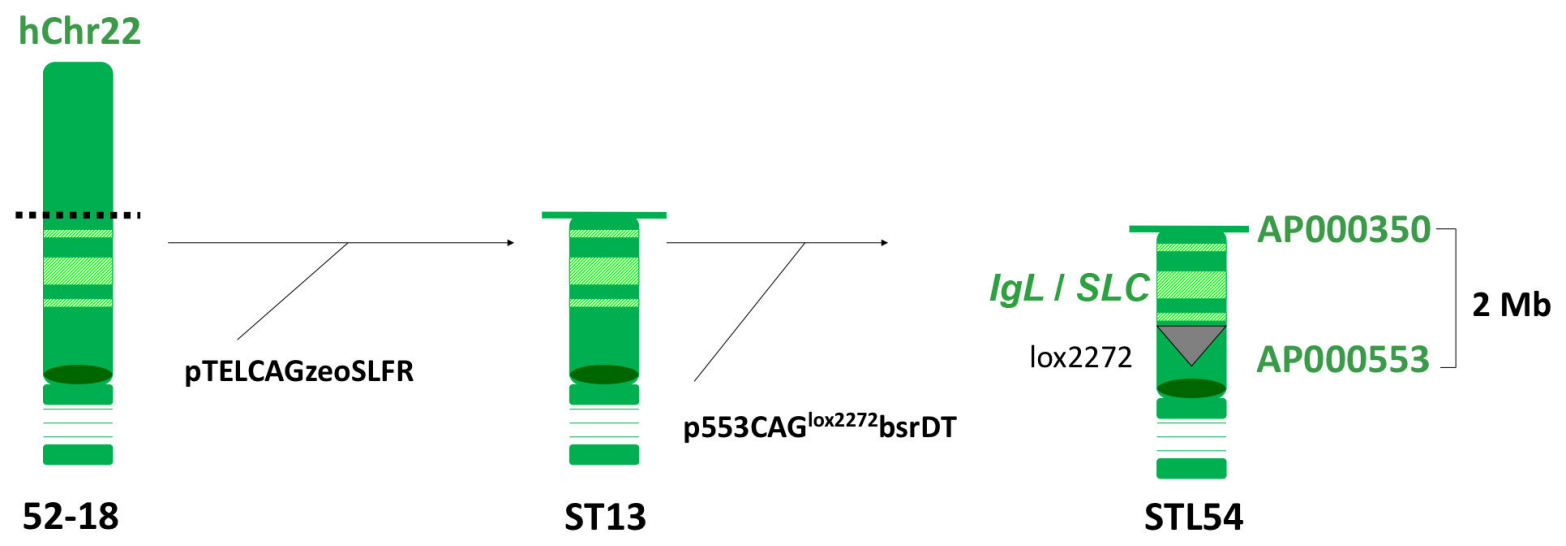
Modification of hChr22. The intact hChr22 carried in a DT40 cell line, 52-18, was first truncated at the AP000350 locus with the targeting vector pTELCAGzeoSLFR. A DT40 clone, ST13, carried the truncated hChr22 was transfected with targeting vector p553CAG^lox2272^bsrDT to integrate the lox2272 and the CAG promoter at the locus AP000553. The distance between AP000350 and AP000553, where the h*IgL* and h*SLC are* located, is about 2 Mb.

To isolate the human chromosome fragment that contains the entire h*IGL* gene cluster and the h*SLC* locus (h*VPREB1*/h*IGLL1*), we set out to modify hChr22 as outlined in Figure **4**. Specifically, a DT40 cell line, 52-18, carrying the intact hChr22 was first truncated at the *AP000350* locus with the targeting vector pTELCAGzeoSLFR and then was further modified with the targeting vector p553CAG^lox2272^bsrDT to integrate the lox2272 and the CAG promoter at the locus *AP000553*. Through extensive genomic PCR screening, a DT40 clone, STL54, was identified to carry the correctly modified hChr22 (Figure 4).

### Modification of hChr2 for HAC Construction

**Figure 5:**
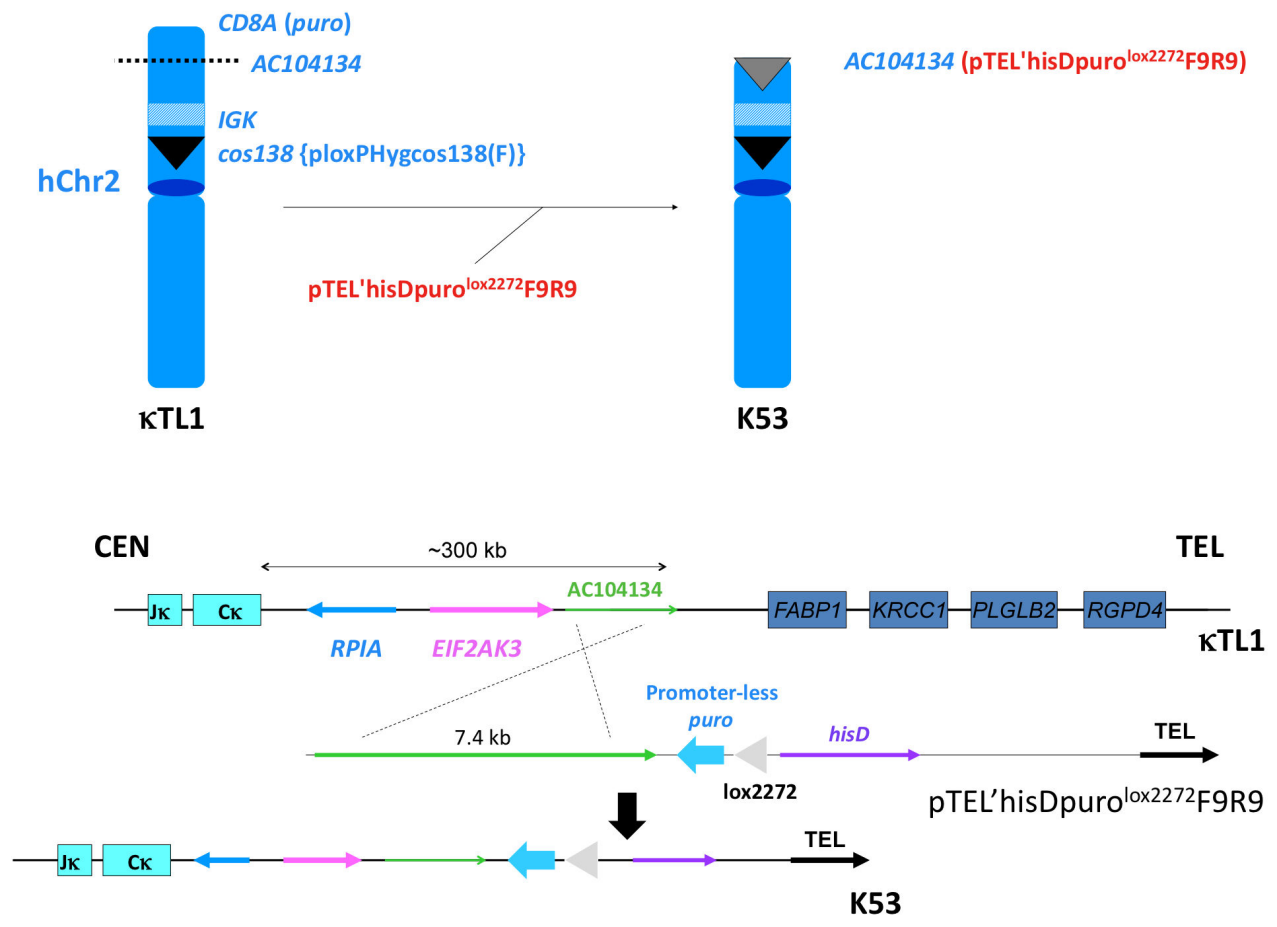
Modification of hChr2. **A** **previously** **engineered** **hChr2** **fragment** **containing** **the** **entire** **h*IGK*** **locus** **in** **a** **DT40** **clone, κTL1** [12]**, was** **further** **modified** **with** **gene** **targeting** **vector** **pTEL’hisDpuro** ^**lox2272**^ **F9R9** **to** **both** **truncate** **the** **hChr2** **fragment** and **integrate** **the** **lox2272** and **the** **promoter-less** ***puro*** **gene** **at** **the** AC104134 **locus**. **The DT40 clone carrying the truncated form of hChr2 was named as K53. The upper panel of the figure shows the structures of hChr2 before and after truncation. The lower panel of the figure depicts the homologous recombination event mediated by the pTEL’hisDpuro^lox2272^F9R9 gene targeting vector.**

We previously engineered a hChr2 fragment containing the entire h*IGK* locus in DT40 cells [12]. We further modified this hChr2 fragment carried by a DT40 clone (named as κTL1) with the targeting vector pTEL’hisDpuro^lox2272^F9R9 to both truncate the hChr2 fragment and integrate the lox2272 and the promoter-less *puro* gene at the *AC104134* locus (Figure 5). Locus *AC104134* is about 300 kb telomeric to the h*IGK* constant region Cκ gene, *IGKC*, and this truncation event deleted all the non-immunoglobulin chromosomal sequences between this locus and the telomere of the p-arm on hChr2. DT40 colonies were screened with genomic PCR (data not shown) for the correctly modified hChr2, and clone K53 was identified and selected for the final HAC construction (Figure 5).

### Translocation between hChr22 and hChr2: Construction of SLK HAC Carrying the h*SLC*, h*IGL* and h*IGK* Loci

**Figure 6:**
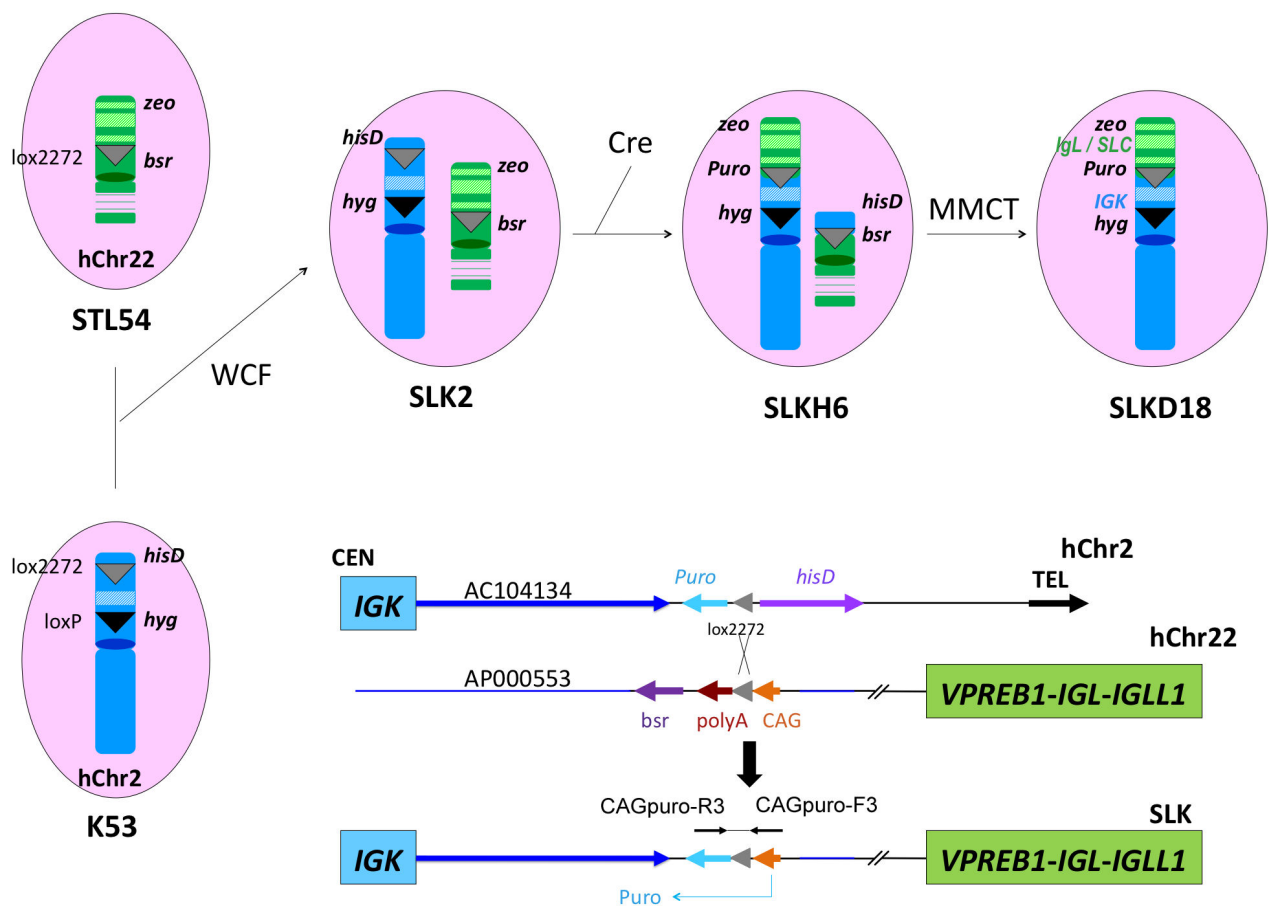
Translocation between hChr22 and hChr2. A DT40 clone (K53) carrying the previously modified hChr2 fragment is fused with a DT40 clone (STL54) carrying the previously modified hChr22 fragment via WCF to generate DT40 hybrid clone SLK2. Transfection of SLK2 by Cre-expression plasmid induces the recombination between the two lox2272 sites, one at the AC104134 locus on the hChr2 and another at AP000553 locus on the hChr22. The translocated chromosome (carried by DT40 clone SLKH6) was transferred to plain DT40 cells by MMCT to generate clone SLKD18. The lower panel of the figure depicts the Cre/loxP mediated DNA recombination event. Note that only the correctly DNA recombination event activates the *puro* gene.

**Figure 7:**
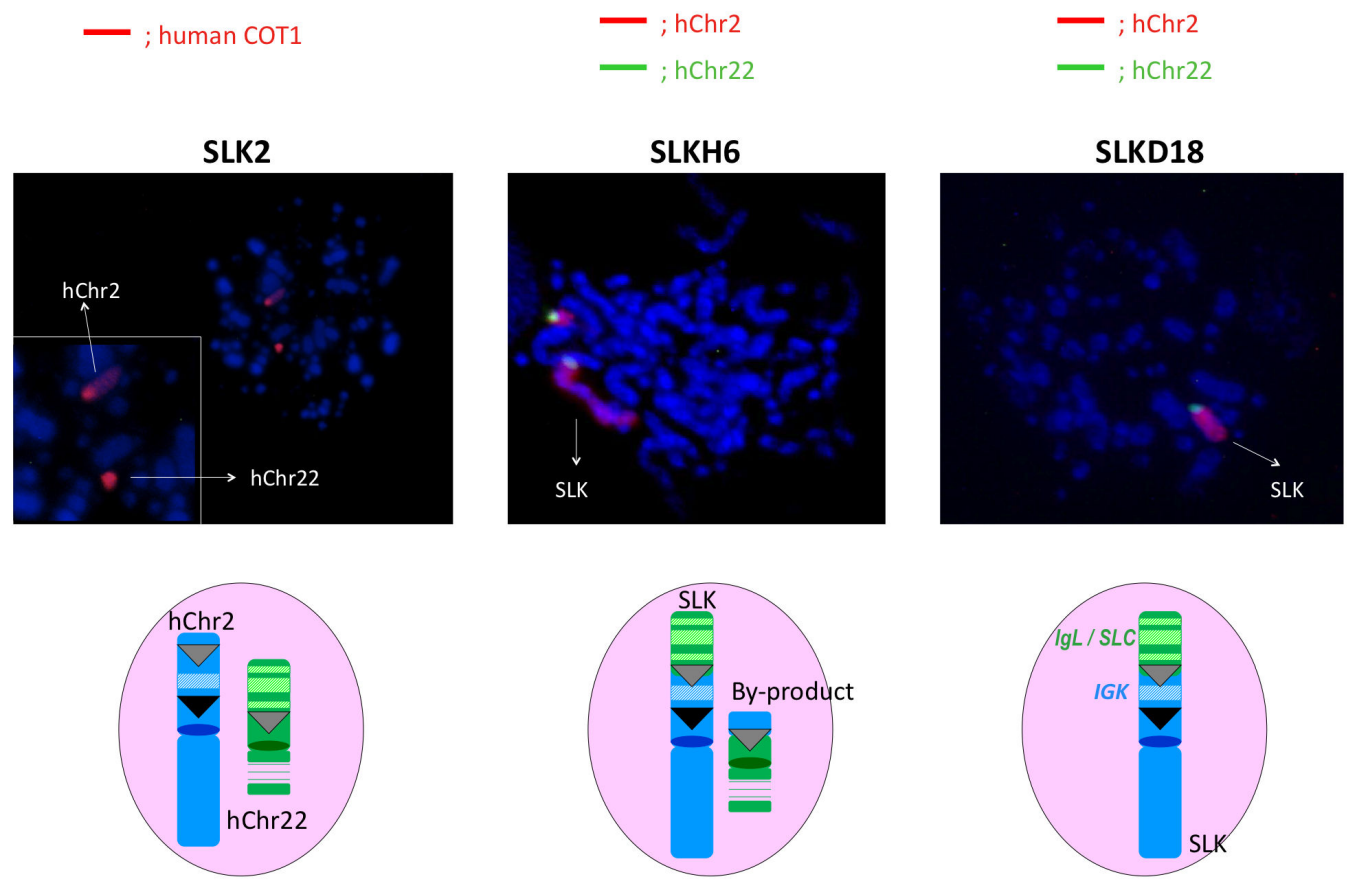
FISH analysis of DT40 clones carrying the corrected modified human chromosomes. For the hybrid DT40 clone SLK2, the presence of both hChr2 and hChr22 was detected by human COT-1 DNA as the probe; for DT40 clones carrying the translocated human chromosomes, a two-color FISH assays were conducted: the hChr2 painting probe was labeled with Rhodamine and the hChr22 painting probe was labeled with Fluorescein.

With the chromosome cloning system we described previously [12], we translocated the h*SLC* and h*IGL* loci on hChr22 to the *AC104134* locus adjacent to h*IGK* locus on hChr2 through Cre/loxP mediated site-specific chromosome recombination (Figure 6). Specifically, a DT40 clone K53 carrying the hChr2 fragment with the previously inserted hisD-lox2272-promoter less *puro* and *hyg*-loxP cassettes, and a DT40 clone STL54 carrying the hChr22 fragment with the previously inserted *bsr* cassette and lox2272, were fused via whole cell fusion (WCF) to generate DT40 hybrid cells. Colonies derived from WCF were screened for the presence of both hChr2 and hChr22 with genomic PCR and FISH by using human COT-1 DNA as the probe as described in Materials and Methods. Clone SLK2 was identified as a positive clone (Figure 7). Finally, to translocate the h*SLC* and h*IGL* loci on hChr22 to the *hIGK* locus at *AC104134* on hChr2 through site-specific recombination, SLK2 was transfected with the Cre expression plasmid to induce the recombination between the two lox2272 sites that were previously inserted at the *AC104134* locus on the hChr2 and *AP000553* locus on the hChr22, respectively (Figure 6). Puromycin resistant colonies (conferred by reconstitution of the CAG promoter-lox2272-*puro* cassette at the translocation site) were screened by genomic PCR with CAGpuro-F3R3 primers, followed by direct sequencing of the PCR product. Clone SLKH6 was identified to contain the correctly generated SLK HAC (Figure 6). We subsequently transferred the SLK HAC from SLKH6 to plain DT40 cells by MMCT. Puromycin resistant and blasticidin S sensitive colonies were screened by extensive genomic PCR (data now shown); candidate clones were further confirmed by two-color FISH using the hChr2 painting probe labeled with Rhodamine and the hChr22 painting probe labeled with Fluorescein (Figure 7). We chose clone SLKD18 for subsequent HAC engineering.

### Construction of cKSL-HACΔ

**Figure 8:**
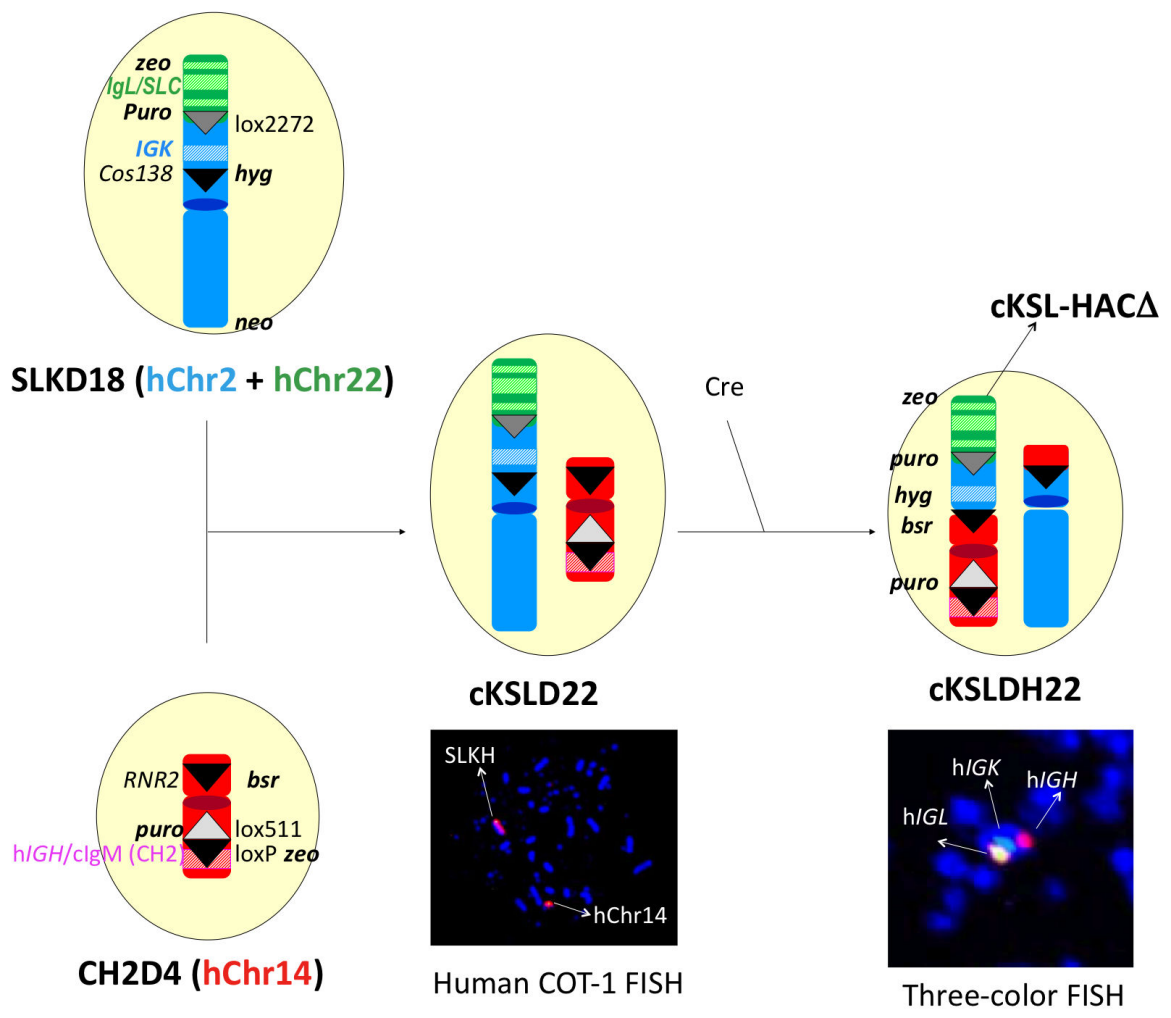
Construction of cKSL-HACΔ. DT40 clones carrying modified hChr14 (CH2D4) and modified hChr2/hChr22 (SLKD18), respectively, were fused by WCF to generate DT40 hybrid clone cKSLD22. The presence of the two modified human chromosomes in cKSLDH22 clone was confirmed by FISH with human COT-1 DNA as the probe. Transfection of cKSLDH22 clone with Cre-expression plasmid induced for site-specific translocation between the two loxP sites at the *cos138* locus on the SLK HAC and another at the *RNR2* locus on the CH2D HAC. The presence of the three human immunoglobulin loci, h*IgH*, h*IgK* and h*IgL*, as a single HAC was confirmed by three-color FISH, with the DNA probes for h*IgH*, h*IgK* and h*IgL* labeled with SpectrumRed, SpectrumGreen, and SpectrumOrange, respectively.

The cKSL-HACΔ was constructed in chicken DT40 cells as outlined in Figure 8. DT40 clones, CH2D4 containing the CH2D HAC and SLKD18 containing the SLK HAC established above were fused by WCF to generate DT40 hybrid clone cKSLD22. After confirming with genomic PCR (data not shown) and FISH with human COT-1 DNA as the probe (Figure 8), the selected clone cKSLDH22 was induced for site-specific translocation between the two loxP sites at the *cos138* locus on the SLK HAC and another at the *RNR2* locus on the CH2D HAC by Cre expression. Cre expression also resulted in the deletion of the floxed CAG promoter-zeo cassette within the cIgM (CH2) domain. Recombinants were enriched by sorting of GFP positive DT40 cells, as GFP expression was conferred by reconstitution of the PGK promoter-loxP-*GFP* cassette at the translocation site (data not shown). cKSLDH22 was finally identified as a DT40 hybrid cell line retaining the cKSL-HACΔ and subjected to extensive genomic PCR (data not shown) and three-color FISH by DNA probes recognizing the h*IGH*, h*IGK* and h*IGL* loci, respectively, for structural confirmation. 

The constructed cKSL-HACΔ was transferred to Chinese hamster ovary (CHO) cells by MMCT to establish CHO-based master cell banks. The structural integrity of the constructed cKSL-HACΔ was further examined by extensive genomic PCR, array comparative genomic hybridization and three-color FISH (data not shown). To our knowledge, this is the first report of construction of an artificial chromosome composed of structurally defined human chromosome fragments from three different chromosomes. 

### Establishing cKSL-HACΔ/DKO Bovine Fibroblast Cell Lines and Tc Cattle Cloning

To clone cKSL-HACΔ/DKO cattle, we established cKSL-HACΔ/DKO bovine fibroblast donor cells by transferring the newly constructed cKSL-HACΔ from CHO cells into the DKO (bIGHM^-/-^ and bIGHML1^-/-^) bovine fibroblast cells via MMCT. Cells from the fibroblast colonies derived from MMCT were used as donors for somatic cell chromatin transfer (CT) as previously described [19]. Cloned embryos developed to the blastocyst stage were singly transferred into recipient cows (one cloned blastocyst to one recipient cow) either for establishing gestation day 40 fetal cell lines or directly for Tc cattle production. Some of the established cell lines were also used for cKSL-HACΔ/DKO cattle cloning. In total, 314 embryo transfers were conducted, with 114 from cell colonies (Col) and 200 from fetal cell lines, and 17 healthy calves were produced (Table 1).

**Table 1:** Pregnancy and live calf data from cell lines used to produce cKSL-HAC/DKO Tc cattle.

**Cell line ID**	**Recipients implanted**	**Pregnant at (%)**			**Live calves (%)**
		**40 d**	**120 d**	**Term**	
Y692 (Col)	74	28 (38)	14 (23)	8 (13)	7 (11)
L289-2	55	12 (22)	9 (16)	4 (7)	2 (4)
598R	85	25 (29)	12 (14)	3 (4)	2 (2)
Y692 (Col)	40	5 (13)	-		
L383	60	33 (55)	23 (38)	8 (13)	6 (10)

**Figure 9:**
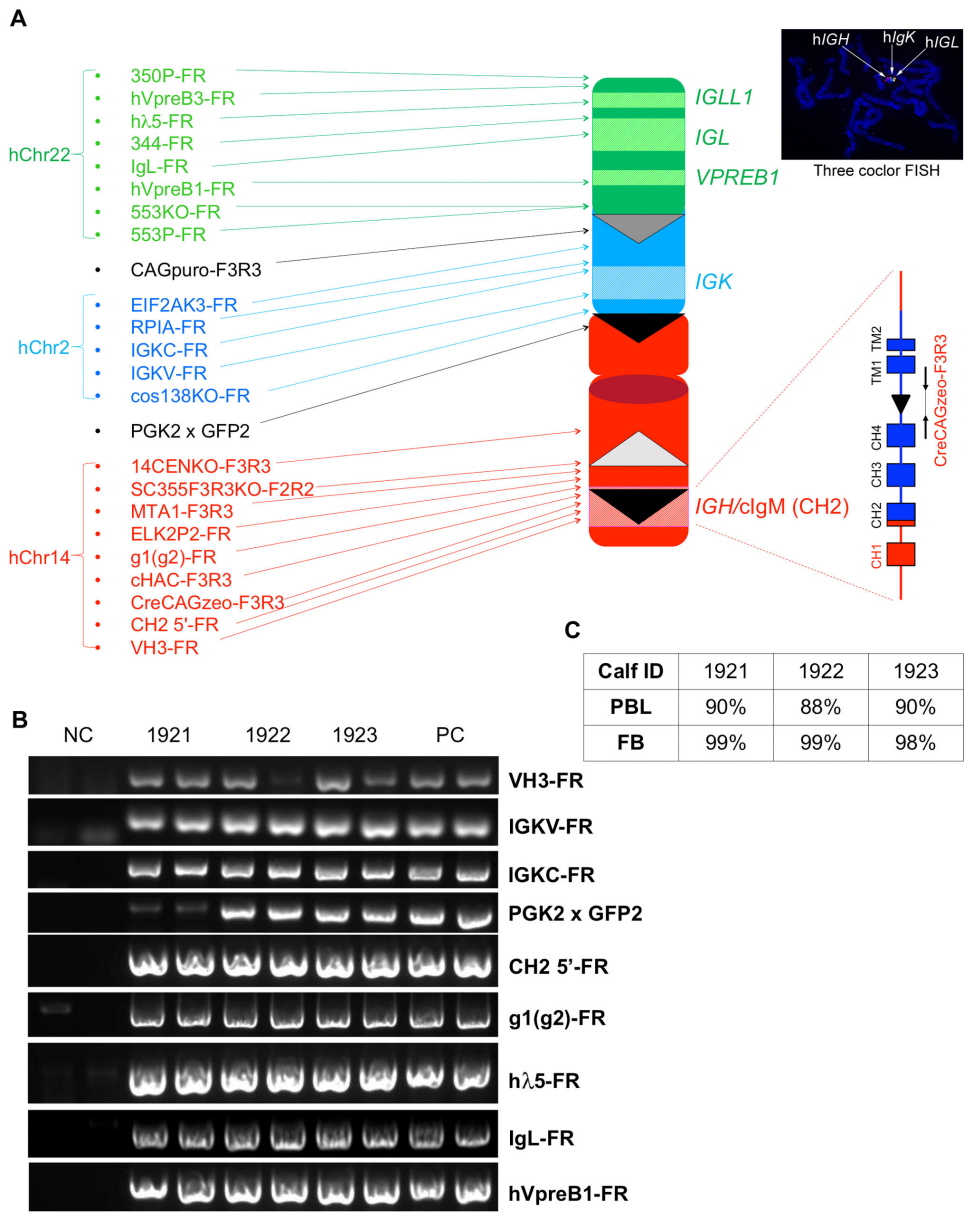
Establish DT40 clones carrying the engineered cKSL-HACΔ. (A), Diagram of cKSL-HACΔ and the locations of PCR primers used to examine cKSL-HACΔ structural integrity. (B), A representative gel image of PCR products for some of the junction points in the cKSL-HACΔ from genomic DNA isolated from PBL of Tc claves 1921, 1922 and 1923. (C) cKSL-HACΔ retention rates in Tc calves 1921, 1922 and 1923 analyzed by FISH. PBL: peripheral blood lymphocytes; FB: fibroblasts.

We confirmed the cKSL-HACΔ/DKO genotype of these Tc calves with extensive genomic PCR with primers amplifying all of the junction points created during the cKSL-HACΔ construction process (Figure 9A and B). Such analyses were performed on the genomic DNAs isolated both from peripheral blood lymphocytes samples and ear biopsies collected from Tc calves (Figure 9B shows a representative gel image of PCR products amplified from the genomic DNAs isolated from peripheral blood lymphocytes). The retention of cKSL-HACΔ and its structural integrity in the produced Tc calves were examined both in the peripheral blood lymphocytes and fibroblasts established from ear biopsies of newborn calves with FISH. The retention rate of cKSL-HACΔ in three of the analyzed calves is shown in Figure 9c. 

### B Cell Development and Human IgG Production Profile in cKSL-HACΔ/DKO Tc Cattle

**Figure 10:**
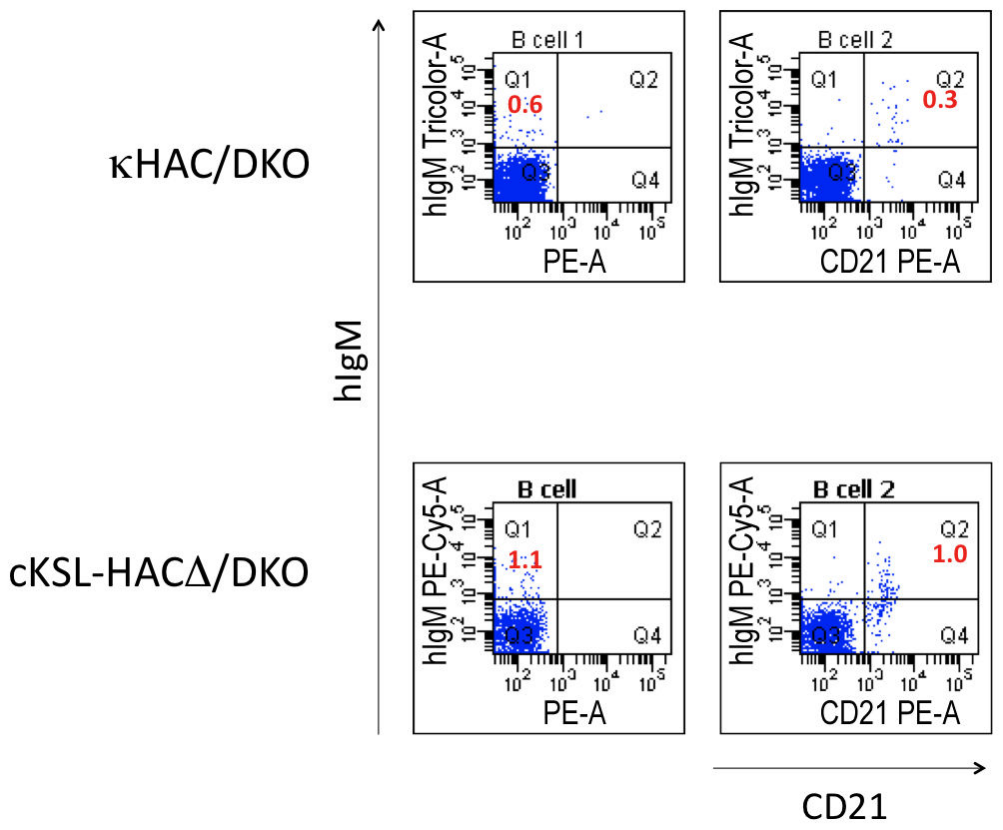
Flow cytometry analysis of B cell development in cKSL-HACΔ/DKO Tc cattle. The percentages of IgM-single positive (IgM^+^) and IgM/CD21-double positive (IgM^+^/CD21^+^) B cells in PBMCs from newborn animals were compared between κHAC/DKO and cKSL-HACΔ/DKO calves.

In order to investigate the impact of HAC modification on B cell development in Tc cattle, we analyzed the peripheral blood mononuclear cells (PBMCs) in newborn animals with flow cytometry. We used anti-hIgM antibody to detect IgM^+^ B cells in cKSL-HACΔ/DKO animals, as the anti-hIgM antibody can still recognize the retained CH 1 domain of the bovinized hIgM. In comparison to the κHAC/DKO animals [12], cKSL-HACΔ/DKO calves demonstrated higher percentage IgM-single positive (IgM^+^) and IgM/CD21-double positive (IgM^+^/CD21^+^) B cells (Figure 10).

**Figure 11:**
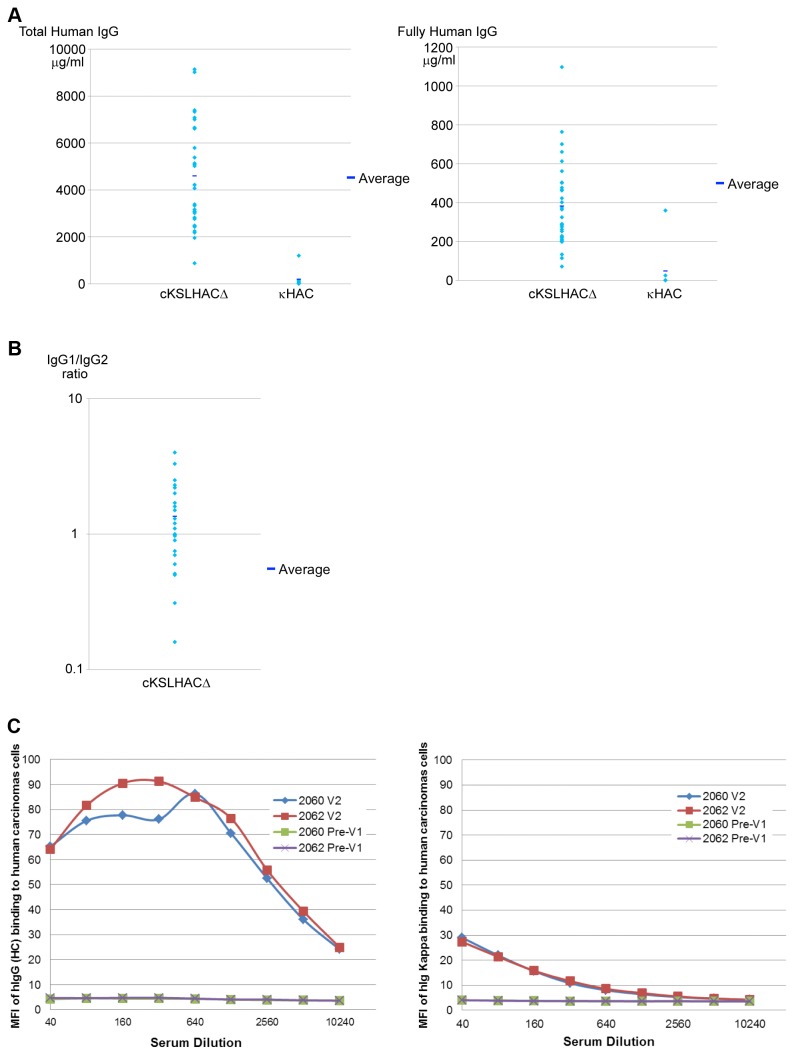
Human IgG production profile in cKSL-HACΔ/DKO Tc cattle. (A) Comparison of average total hIgG (left panel) and fully hIgG (hIgG/hIgκ) concentrations in the sera of κHAC/DKO and cKSL-HACΔ/DKO calves at 5-6 months of age. (B) IgG subclass distribution in the plasma of cKSL-HACΔ/DKO calves. (C) Mean fluorescence intensity (MFI) of tumor cells stained with the sera from immunized cattle. Left panel shows the MFI with anti-hIgG antibodies (measuring total hIgG); right panel shows the MFI with anti-hIgκ antibodies (measuring fully hIgG/hIgκ).

We measured the serum concentrations of total hIgG, i.e. all the hIgG regardless of whether it pairs with human lambda or kappa (hIgG/hIgλ/κ) light-chains or bovine lambda or kappa (hIgG/bIgλ/κ) light-chains, as well as fully hIgG (hIgG/hIgλ/κ only), when the Tc calves reached to 5-6 months of age. Even though the total hIgG levels varied among these Tc calves, the overall total hIgG production was found to be dramatically improved compared to the κHAC/DKO Tc animals, at levels comparable to the physiological hIgG levels found in healthy humans (Figure 11A). Nevertheless, the average level of fully hIgG (hIgG/hIgλ/κ) produced by cKSL-HACΔ/DKO calves was about 0.383 g/l which is only about 8.5% of the total hIgG. This is not unexpected, as the bovine immunoglobulin light-chain genes, lambda and kappa, were not genetically knocked out in such Tc cattle, the expression of human light-chain loci on the HAC would be easily overwhelmed by the endogenous bovine light-chain locus resulting in the produced hIgG mostly to be chimeric (hIgG/bIgk/l). We also analyzed the IgG subclass distribution in the plasma of such Tc animals and showed that IgG1 is the dominant subclass (hIgG1/hIgG2 ratio >1) which is similar to what is observed in the plasma of healthy humans (Figure 11B). Therefore, we produced Tc cattle expressing physiological levels of hIgG with a subclass distribution profile similar to that of healthy humans.

### cKSL-HACΔ/DKO Tc Cattle Produce High Titer hIgG against Tumor Antigens

As our ultimate goal is to produce high titer antigen-specific hpAbs from the Tc cattle, we conducted an immunization study by immunizing two Tc cattle with a human oral squamous cell carcinoma cell line as an antigen. To evaluate antibody titers specific to the tumor cells, we collected sera from each of the immunized Tc cattle at 14 days post second vaccination (V2) and performed flow cytometry-based immunofluorescence assay. As shown in Figure 11c, Tc calves readily responded to immunization and generated high hIgG titer against human oral squamous cell carcinomas post V2 as demonstrated by the mean fluorescence intensity (MFI) of tumor cells stained with the sera from immunized cattle. Furthermore, when MFI was measured by using anti-human kappa antibodies (to detect fully hIgG staining of the tumor cells, see Materials and Methods), measurable MFI was detected even after a 10,000-fold dilution of the sera. However, the titer against human tumor cells from fully hIgG is significantly lower than that from total hIgG. This was expected due to the fact that bovine immunoglobulin light-chain genes have not been genetically inactivated in these Tc cattle and only roughly 8% fully hIgG is produced. 

## Discussion

There are numerous technical challenges in producing fully hpAbs totally independent of the human immune system. First, as the human immunoglobulin genes and gene segments exist as gene clusters spanning large regions on three chromosomes, the only feasible way currently of delivering such a large size of human genomic DNA into a biological system for hpAbs production is through the engineering of HACs that comprise all or most of the human Ig genes. We report here the successful engineering of a HAC comprising the entire hIg gene repertoire that reside on three different human chromosomes, i.e. the IgH locus from hChr14, the Igk locus from hChr2, and the Igλ and surrogate light-chain loci from hChr22. The second challenge for fully hpAbs production by a recombinant system is to have a biological mechanism to efficiently tap into the genetic information provided by the immunoglobulin gene repertoires for generating the seemingly unlimited diversity of hpAbs. The mammalian humoral immune system is currently the only conceivable biological system that has such functionality. Therefore, we set out to develop a Tc animal system for fully hpAbs production. We chose bovine for the large body size, which makes it feasible to collect large volumes of plasma, a prerequisite for producing large amounts of therapeutic hpAbs. 

However, our initial success in producing antigen-specific fully hpAbs in the Tc cattle system was limited by the relatively low levels of total hpAbs production and the low level of fully hpAbs production [12]. In this report, we addressed this issue by improving the pre-BCR function through incorporating the human surrogate light-chain genomic locus into the HAC and by bovinizing the HAC in the hIgM domain that is involved in interacting with bovine Igα and Igβ signal molecules in the pre-BCR complex. Our results showed that restoring intra-species interactions between proteins in pre-BCR had a positive impact on Tc bovine B cell development as demonstrated by the increased development of IgM^+^/CD21^+^ B cells in newborn Tc calves (Figure 10). Very importantly, a dramatic improvement in total hIgG production was shown in these Tc calves as compared to our previously reported κHAC/DKO Tc animals, likely due to improved B cell development. Finally, we demonstrated that Tc cattle developed here are responsive to antigen immunization and generate high titer antigen-specific human polyclonal antibodies (Figure 11). Therefore, we developed an improved Tc bovine system to produce large quantities of hpAbs.

There are apparently some limitations that still exist in this Tc bovine system, as the endogenous bovine immunoglobulin light-chain genes are intact and 80% of total serum hIgG produced is chimeric composing the hIgG heavy and bIg light-chains. While such chimeric hIgG are fully functional in recognizing antigens (our unpublished data) and are likely much less immunogenic than fully animal derived antibodies for therapeutic applications, it is obvious that fully human antibodies are more desirable as a biotherapeutic. This is especially true for long-term, repetitive treatments. Therefore, we have recently knocked out the bovine lambda light genes by deleting the entire bovine lambda light-chain gene cluster (manuscript in preparation) and are in the process of knocking out the bovine kappa light-chain genes. Accompanying our efforts to genetically improve our Tc bovine system, we also have developed robust immunization protocols in Tc bovine for producing high titer immunogen-specific fully hpAbs. The fact that high titer antigen-specific hpAbs can be produced from Tc animals through hyper-immunization will offer great advantages over the human plasma derived hpAbs in that the titers to certain pathogens from human plasma derived hpAbs tends to be low and sources of convalescent plasma are often difficult to obtain. 

In summary, our improved Tc bovine system for the production of fully hpAbs has further advanced achieving the goal of producing large quantities of therapeutic hpAbs as an alternative for plasma derived hpAbs.

## Materials and Methods

### Ethics statement

The animal protocols contained in the study were approved by the Hematech (previous name of Sanford Applied Biosciences) Institutional Animal Care and Use Committee (IACUC) (USDA Research Facility 46-R-0008, OLAW #A4438-01, AAALAC #001114). Care of all vertebrate animals is subject to regular review by the IACUC and complies with Animal Welfare laws and regulations of the United States. Periodic health evaluations (blood profile, weight, etc.) are made by Veterinary Service to ensure that Tc cattle are healthy. All Tc bovine receive adequate housing, feed, access to water and bedding. Daily observations are made by herdsmen to ensure that appropriate standards of animal care are being met. Sanford Applied Biosciences uses the *Guide* *for* *the* *Care* *and* *Use* *of* *Laboratory* *Animals* and the *Guide* *for* *the* *Care* *and* *Use* *of* *Agricultural* *Animals* *Used* *in* *Agricultural* *and* *Research* *Teaching* for animal care standards. Animal care and use and facilities are inspected by the IACUC on a semiannual basis. Animals may have to be euthanized due to unfortunate events like terminal illness and trauma. Methods of euthanasia must follow the guidelines given by the American Veterinary Medical Association (AVMA) *2013*. These are acceptable methods by the *AVMA* and they have been approved by the IACUC. All protocols and procedures used in the Animal Care and Use Program that may cause discomfort, distress and pain, and injury are reviewed by the IACUC for appropriate management practices. Analgesic, anesthetics and tranquillizing drugs are used when appropriate under supervision of Veterinary Services. Veterinarians and herdsmen use cattle chute for restraining when needed. Cattle are not restrained for more than four hours per day. 

### Genomic library

Genomic DNA isolated from primary bovine fibroblast cell lines established from Holstein dairy cattle was used to construct the bovine genomic library. Each λ phage-based genomic library was constructed using λFIX II vector through a custom library construction service (Lofstrand Labs Ltd., Gaithersburg, MD). Library screening and λ phage DNA extraction/purification was done as described previously [12]. 

### Construction of gene targeting vectors

pTEL’hisDpurolox2272F9R9: A previously described plasmid pTELpuro [12] was modified by replacing the *puro* gene with the *hisD* gene, *Eco* RI site with *Srf* I, and *Spe* I site with *Pme* I site to generate plasmid pTEL'hisDPm. A pPURlox2272 plasmid was constructed by cloning the double strand lox2272-containing oligo DNA (Oligo DNA pair 1) into HindIII site of plasmid pPUR (BD Bioscience Clontech, Mountain View, CA). In parallel, a 7.3kb homologous arm (amplified by using a PCR primer pair, kD-F9 and kD-R9, in 40 cycles of 98 °C for 10 s and 68 °C for 9 min) was subcloned into *Bam* HI site of plasmid pTEL’hisDpurolox2272. Then the *Bam* HI fragment from the pPURlox2272 was blunted and cloned to *Pme* I site of the pTEL'hisDPm to generate the pTEL’hisDpurolox2272F9R9 vector. 

pTELCAGzeoSLF2R2: plasmid pTELpuro was modified by converting the *Eco* RI site to *Pme* I and by replacing the puro gene with CAG*zeo* gene to generate vector pTELCAGzeo(Sr)Pm. Then the second homologous arm (7.4kb) (amplified by using a PCR primer pair, SL-F2 and SL-R2, in 40 cycles of 98 °C for 10 s and 68 °C for 9 min) was cloned into *Bam* HI site of the plasmid pTELCAGzeo(Sr)Pm to generate pTELCAGzeoSLF2R2. 

p553CAGlox2272BsrDT: A previously described targeting vector pHCF2loxPHyg [17] was modified by replacing the homology arm sequence of the *HCF2* gene with that of the *AP000553* (amplified by using a PCR primer pair, 553-F3 and 553-R3, in 40 cycles of 98 °C for 10 s and 68 °C for 15 min) to generate p553loxPHyg(F). This plasmid was Not I-digested and self-ligated to remove loxPHyg cassette, followed by cloning of *dT-A* fragment into *Srf* I site. In parallel, pDRIVE-CAG (InvivoGen, San Diego, CA) was modified by replacing the lacZ fragment (*Bsr* GI-*Eco* RI) with the loxP-containing oligo DNAs (Oligo DNA pair 2). Then *Sda* I-*Swa* I fragment was liberated from this plasmid and cloned into *Pst* I/*Sma* I-digested pBluescript SK^−^ (Stratagene, La Jolla, CA) to generate pCAGloxP. The loxP sequence was then replaced by the lox2272-containing sequence that was generated after annealing Oligo DNA pair 3. Then the *bsr* gene was added to *Spe* I site to generate pCAGloxP2272bsr. Finally, the *Not* I-*Kpn* I fragment (CAG-lox2272-polyA-*bsr*) was cloned into *Not* I site to complete p553CAGlox2272BsrDT. 

pSC355CAGlox511hisDDT: a genomic DNA fragment (10.2 kb) as one of the homologous arms was amplified by using a PCR primer pair, SC355-F3 and SC355-R3, in 40 cycles of 98 °C for 10 s and 68 °C for 15 min. This PCR product was cloned into *Spe* I site of a plasmid pBluescript where the *Kpn* I site was converted to *Srf* I site to generate pSC355F3R3. The pCAGloxP plasmid was similarly modified by replacing the loxP sequence with the lox511-containing sequence (Oligo DNA pair 4) and by adding the *hisD* gene to *Spe* I site to generate pCAGlox511hisD. The *Not* I-*Kpn* I fragment (CAG-lox511-polyA-*hisD*) from pCAGlox511hisD was cloned into the *Eco* RV site of pSC355F3R3. Finally, the *dT-A* cassette was cloned into *Not* I site to complete pSC355CAGlox511hisDDT. 

p14CEN(FR)hygpurolox511DT: a genomic DNA fragment (10.2 kb) for another homologous arm was amplified by using a PCR primer pair, 14CEN-F and 14CEN-R, in 40 cycles of 98 °C for 10 s and 68 °C for 15 min. This PCR product was cloned into *Bam* HI site of a plasmid pBluescript where the *Kpn* I site was converted to *Pme* I site to generate p14CEN(FR). The modified lox511-containing oligoDNA (Oligo DNA pair 5) was cloned into *Hin* dIII site of a plasmid pPUR (BD Bioscience Clontech, Mountain view, CA) to generate plasmid pPURlox511. The *Bam* HI fragment from the pPURlox511 was cloned to *Bam* HI site of pBluescript SK^−^ (Stratagene, La Jolla, CA), followed by cloning of the *hyg* gene to *Eco* RV, to generate pHygPurolox511. The *Not* I-*Kpn* I fragment (*puro*-lox511-*hyg*) was cloned into the *Hpa* I site of p14CEN(FR). Finally, the *dT-A* cassette was subcloned into *Pme* I to complete p14CEN(FR)hygpurolox511DT. 

pRNR2loxPbsrDT: the previous vector pRNR2loxPbsr [17] was modified to construct the pRNR2loxPbsrDT by simply adding the *dT-A* cassette. 

pCH1CAGzeo(R)DT(F): a genomic λ phage library was constructed from CHO cells containing the κHAC using λFIX II vector through a custom library construction service (Lofstrand Labs Ltd., Gaithersburg, MD). The genomic library was screened for h*IGHM* constant region by using a probe that was a PCR product (with PCR primer pair, hCμ-FR). A 1.7 kb of *Pml* I fragment from one of the positive phage clones was cloned into *Sma* I site of pBluescript to generate pCH1S (F). Then a 1 kb of *Sac* I-*Pml* I bovine *IGHM* genomic fragment from plasmid pBCμAY37-95 (a clone containing *IGHM* bovine genomic fragment derived from lambda phage based genomic library [12] was subcloned into *Pst* I site of the pCH1S (F) to generate pCH1SSP (F). A 7.4 kb of the *Sma* I-*Eco* RI fragment from another positive phage clone was cloned into *Eco* RV/*Eco* RI-digested pCH1SSP (F) to generate pCH1SL. In parallel, a 3.5 kb of *Sac* I fragment from pBCμAY37-95was cloned into pBluescript and then the *Xho* I fragment of floxed CAGzeo was cloned into *Van91* I site to generate pmAYSazeo (F). The *Sac* I fragment from the pmAYSazeo (F) was then cloned into blunted *Eco* RI site of pCH1SL resulting in pCH1zeo (F). As a final step, the *dT-A* cassette was cloned into *Not* I site of the pCH1zeo (F) to complete pCH1CAGzeo(R)DT(F). 

pCH2CAGzeoDT: an annealed oligo DNA pair, SeSp, which contains human and bovine junction region at CH2 flanked by SexAI and Sph I site, was cloned into blunted *Pst* I site of pBluescript. From the pBCμAY37-95, a 2 kb of *Sph* I-*Bam* HI fragment was cloned into *Sph* I-*Bam* HI site of the plasmid generated by cloning SeSp into the Pst I site, generating pmAYSpB. Similarly, a 2 kb of *Bam* HI-*Pml* I fragment from the pBCμAY37-95 was cloned into *Bam* HI-*Pme* I site (converting the original *Spe* I site) resulting in pmAYSpBPml. A 0.6 kb of *Eco* RI-*Sex* AI fragment from the above phage clone was cloned into *Eco* RI-*Sex* AI site of the pmAYSpBPml to generate pRISe. Then the floxed CAGzeo was cloned into *Van91* I site of the pRISe to generate pRISeCAGzeo (R), of which *Not* I site was converted to *Eco* RI site, generating pRISeCAGzeoE. Meanwhile, 1.7 kb of *Pml* I fragment from the above clone 4 was subcloned into *Sma* I site of pBluescript of which *Eco* RV site was converted to *Mlu* I site, generating pCH2S (F). 6.6 kb of *Mlu* I-*Eco* RI fragment from the above clone 1 was cloned into *Mlu* I-*Eco* RI of the pCH2S (F), generating pCH2LS. Then, the *Eco* RI fragment from the pRISeCAGzeoE was subcloned into *Eco* RI site of the pCH2LS, generating pCH2CAGzeo (F). As a final step, the *dT-A* cassette was subcloned into *Not* I site of the pCH2CAGzeo (F) to complete the pCH2CAGzeoDT. 

### Transfection of chicken DT40 cells for HAC vector construction

HAC vector construction was carried out as previously described [12,17,20]. Briefly, DT40 (Japan Collection of Research Bioresources, #9130, Japan) cells containing each hChr fragment were electroporated by using Gene Pulser II (550 V, 25 μF) with ~25 μg of each targeting vector. DT40 cell colonies were selected by the appropriate drugs based the experimental design. The concentrations of drugs used are the following: G418 (2 mg/ml), puromycin (0.5 μg/ml), hygromycin B (1.5 mg/ml), blasticidin S (15 μg/ml), histidinol (0.5 mg/ml) or zeocin (1 mg/ml). Genomic DNAs isolated from selected cell colonies were subjected to PCR screening to identify the colonies with the correct gene targeting events.

### Micro-cell Mediated Chromosome Transfer

MMCT was done with each HAC vector as described previously [12,17,20].

### Genomic PCR and RT-PCR

These analyses were implemented as previously described [12,17,20,21]. The PCR primer sequences used are listed in Table S1 (Oligo DNA information). All the PCR products were resolved on 0.8 % agarose gels for analysis. Primer sequences are available from Table S1 (Oligo DNA information).

### CGH analysis

Array probe design, chip hybridization, and data analysis were performed by Roche NimbleGen (www.nimblegen.com). 

### FISH analysis

Human COT-1 FISH and hChr-specific multi-color FISH were performed as previously described [12]. To specifically stain the h*IGH*, h*IGK* and h*IGL* loci, probes were synthesized from DNA derived from BAC clones RP11-417P24, RP11-316G9 and RP11-22M5 (MBL-EBI and Sanger Institute: http:// www.ensembl.org/index.html), respectively. 

### Somatic cell nuclear transfer

Cloned fetuses and calves were produced using chromatin transfer procedure as described previously [19].

### Flow cytometry analysis

Flow cytometry analysis on B cell development in newborn Tc calves was performed as previously described [12], except for some of the detection antibodies used. To detect surface hIgG on Tc bovine B cells, goat anti-hIgG (Life Technologies, Grand Island, NY) directly labeled with AF 488 was used. To label surface hIgκ or hIgλ on Tc bovine B cells, mouse anti-hIgκ antibody directly labeled with PE (Biolegend, San Diego, CA) or mouse anti-hIgλ antibody directly labeled with PE (Southern Biotech, Birmingham, AL) was used. To label surface bIgλ or bIgκ on the B cells, mouse monoclonal anti-bIgλ (in-house clone 132D7) or mouse monoclonal anti-bIgκ (in-house clone 132B10) followed by Zenon mouse IgG1PE labeling (Life Technologies, Grand Island, NY) were used. Staining was done by a standard protocol and then analyzed by FACSAria flow cytometer (BD Biosciences, San Jose, CA). Briefly, 10^6^ cells were incubated with primary antibody on ice for 20 min. After washing twice, the cells were incubated with appropriate secondary antibody for 20 min on ice if the primary antibody is not directly labeled with a fluorochrome. Finally, the cells were washed followed by re-suspend in FACS stain media for reading.

### ELISA

Total hIgG ELISA assay was performed as previously described [12]. For fully hIgG/hIgκ or hIgG/hlgλ detection, goat anti-hIgκ affinity-purified or goat anti-hlgλ affinity-purified (Bethyl, Montgomery, TX) as a capture and goat anti-hIgG Fc-HRP (Bethyl, Montgomery, TX) as a detection antibody were used. For hIgG/bIgκ detection, mouse monoclonal anti-bIgκ (in-house clone 132B10) as a capture and mouse anti-hIgG Fcγ-HRP (Jackson immunoResearch, West Grove, PA) as a detection antibody was used. For detection of hIgG1 or hIgG2, mouse anti-hIgG1 Fc or mouse anti-hIgG2 Fc (Hybridoma Reagent Laboratory, Baltimore, MD) as a capture and mouse anti-hIgG HRP (Southern Biotech, Birmingham, AL) as a detection antibody were used.

### Immunization of human oral squamous cell carcinoma to cKSL-HACΔ/DKO calves.

cKSL-HACΔ/DKO calves were immunized with X-ray-irradiated human oral squamous cell carcinoma (DSMZ) antigen at 2 x10^8^ cells/dose formulated with Montanide ISA 25 adjuvant (Seppic, Puteaux, France) as water-in-oil emulsion plus Quil A (Accurate Chemical & Scientific Corp, Westbury, NY) as immune stimulant. The Tc calves were immunized two times at 3-week intervals (primary immunization followed by the booster after 3 weeks). Vaccine was administered by intramuscular injection in the neck region. Serum samples were collected as previously described [5] before each immunization (V1 and V2) and 10 days and 14 days after each immunization for antibody titer analysis. Anti-human oral squamous cell carcinoma antibody titers were determined by flow cytometry analysis.

### Measurement of anti-human carcinoma cell hIgG/hIgκ titer in Tc animal sera by flow cytometry

Sera collected from Tc calves immunized with human carcinoma cells were used as the primary antibody to stain the human carcinoma cells. Pre-immune Tc calf serum (V1, day 0) was used as the negative controls. AF488-conjugated goat anti-hIgG Fc (Invitrogen, Grand Island, NY) at 1:80 dilution and PE-conjugated mouse anti-hIgκ (Biolegend, San Diego, CA) at 1:8 dilution were used to detect bound hIgG/hIgκ antibody. The assay was performed in PBS supplemented with 4 % horse serum, 0.1% sodium azide and 2 mM EDTA. The results were expressed as mean fluorescence intensity (MFI) as measured by FACSAria flow cytometer (BD Biosciences, San Jose, CA).

## Supporting Information

Table S1 **Oligo DNA information.**
(XLSX)
