# Correction: Physiological Level Production of Antigen-Specific Human Immunoglobulin in Cloned Transchromosomic Cattle

**DOI:** 10.1371/annotation/82d9a0e0-a898-4b29-83d7-9daf6f5c384c

**Published:** 2014-01-06

**Authors:** Akiko Sano, Hiroaki Matsushita, Hua Wu, Jin-An Jiao, Poothappillai Kasinathan, Eddie J. Sullivan, Zhongde Wang, Yoshimi Kuroiwa

The Funding Statement is incorrect. The Funding Statement should read: "This work was financed by internal funding of Hematech, Inc., which no longer exists and no longer has financial interest in the work. Hematech has transferred all of its business to Sanford Applied Biosciences, LLC. There are no current external funding sources for this study. Financial interest in this work is currently owned by Sanford Applied Biosciences who did have a role in the decision to publish as well as the preparation of the manuscript. While it is considered beneficial to the interests of Sanford Applied Biosciences to publish this work, this consideration played no role in the study design, data collection and analysis and preparation of the manuscript. This is evidenced by the inclusion of now independent authors with no affiliation to Sanford Applied Biosciences." 

